# Assessment of autonomous nerve system through non-linear heart rate variability outcomes in sedentary healthy adults

**DOI:** 10.7717/peerj.10178

**Published:** 2020-11-02

**Authors:** Gines Navarro-Lomas, Alejandro De-la-O, Lucas Jurado-Fasoli, Manuel J. Castillo, Pedro Femia, Francisco J. Amaro-Gahete

**Affiliations:** 1 EFFECTS-262 Research Group, Department of Physiology, University of Granada, Granada, Spain; 2 Department of Statistics, University of Granada, Granada, Spain

**Keywords:** HRV, Stress score, Sympathetic/parasympathetic ratio, Kubios, Poincare plot

## Abstract

**Background:**

Heart rate variability (HRV) is a psycho-physiological phenomenon with broad health implications. Different data analysis methods have been used to assess the autonomic nervous system activity, but the validation of new indexes that accurately describe its balance through non-invasive methods (i.e., HRV analysis) is of clinical interest. This study aimed: (i) to evaluate the association of the Stress Score (SS) and the Sympathetic/Parasympathetic Ratio (S/PS) with time domain and frequency domain analysis of HRV, and (ii) to set reference values of SS and S/PS in sedentary healthy adults.

**Methods:**

A total of 156 sedentary healthy adults (38.4 ± 15.57 years old, 81 women), aged were involved in this study. HRV was measured for 15 min in a supine position at rest. SS and S/PS were calculated from the non-linear HRV analyses based on Poincare Plot.

**Results:**

Stress Score showed a non-linear negative power-law relationship with SDNN (β = −0.969; *R*^2^ = 0.963; *P* < 0.001), RMSSD (β = −0.867; *R*^2^ = 0.722; *P* < 0.001), high frequency (β = −0.834; *R*^2^ = 0.752; *P* =< 0.001), low frequency (β = −0.627; *R*^2^ = 0.330; *P* < 0.001), SD1 (β = −0.867; *R*^2^ = 0.722; *P* < 0.001) and SD2 (β = −1.000; *R*^2^ > 0.999; *P* < 0.001). There was observed a negative cubic relationship between SS with PNN50 (β = −1.972; *R*^2^ = 0.644; *P* < 0.001). A linear regression model was conducted between SS with Ratio Low/High Frequency (β = 0.026; *R*^2^ < 0.001; *P* = 0.750). Non-linear power-law regression models were built between S/PS and SDNN (β = −0.990; *R*^2^ = 0.981; *P* < 0.001), RMSSD (β = −0.973; *R*^2^ = 0.939; *P* < 0.001), high frequency (β = −0.928; *R*^2^ = 0.970; *P* < 0.001), low frequency (β = −2.344; *R*^2^ = 0.557; *P* < 0.001), SD1 (β = −0.973; *R*^2^ = 0.939; *P* < 0.001) and SD2 (β = −0.611; *R*^2^ = 0.908; *P* < 0.001). A non-linear negative regression model was built between S/PS and PNN50 (β = −3.412; *R*^2^ = 0.868; *P* < 0.001). A linear regression model was conducted between S/PS and SD2/SD1 (β = 0.075; *R*^2^ = 0.006; *P* < 0.001).

**Conclusion:**

Our results support the use of SS as a sympathetic activity marker, and S/PS as an indicator of the sympathetic and parasympathetic activity of the autonomic nervous system in sedentary healthy adults.

## Introduction

Heart Rate Variability (HRV) describes the time differences between successive heart beats, and it is commonly assessed by measuring electrocardiographic RR intervals (Camm et al., 1996). According to Dantas et al. (2018), HRV is a psycho-physiological phenomenon with broad implications, that include physiological, neuro-psychological, pathological, environmental and lifestyle issues, in addition to non-modifiable factors such as age or gender (Ernst, 2017; Fatisson, Oswald & Lalonde, 2016). HRV is an accepted non-invasive method commonly used to describe the influence of the Autonomic Nervous System (ANS) on heart function (Almeida-Santos et al., 2016; Germán-Salló & Germán-Salló, 2016; Sztajzel, 2004). Increased HRV is linked to reduced stress and good health, while decreased HRV is associated with chronic diseases and high cardiovascular risk (Tsuji et al., 1996).

Heart Rate Variability characteristics can be determined by three different data analysis methods: (i) Time-Domain analysis, that includes, among others, standard deviation of RR intervals (SDNN), root mean square of successive differences (RMSSD), and percentage of successive RR intervals that differs in more than 50 ms (PNN50); (ii) Frequency-Domain analysis, that includes High Frequency (absolute values of power in 0.15–0.4 Hz band in ms), low frequency, (absolute values of power in 0.04–0.15 Hz band in ms) and Ratio Low/High Frequency (calculated as a quotient between Low and High Frequency) (Camm et al., 1996); and (iii) Non-linear analysis, an approach which aims to quantify the structure and complexity of RR interval time series (Electrophysiology TF of the ES, 1996).

The HRV signals are non-stationary and non-linear in nature. The analysis of HRV dynamics by methods based on chaos theory and non-linear system theory is established on observations clearly indicating that the mechanisms involved in cardiovascular regulation likely interact with each other in a non-linear manner (Germán-Salló & Germán-Salló, 2016). Poincare Plot is widely considered as a non-linear method to analyse HRV (Tulppo et al., 1996) although there is still controversy about this regard (Brennan, Palaniswami & Kamen, 2001). The RR intervals are represented against the previous one in a two-dimensional dispersion plot, showing a qualitative picture of the variations between RR intervals (Woo et al., 1992) and a quantitative HRV outcome (Brennan, Palaniswami & Kamen, 2001). SD1 is defined as the standard deviation orthogonal to the line of identity in Poincare Plot and SD2 is defined as the standard deviation along the line of identity (Shaffer & Ginsberg, 2017). SD1 is directly associated with parasympathetic activity (Hoshi et al., 2013). SD2, although is less well defined, seems to be inversely proportional to sympathetic activity (Naranjo-Orellana et al., 2015). Some studies have reported an association of SD2 with sympathetic and parasympathetic activity (De Vito et al., 2002), while others have shown a negative strong association between SD2 and sympathetic activity (Naranjo-Orellana et al., 2015; De la Cruz Torres, Lopez & Naranjo-Orellana, 2008).

Naranjo-Orellana et al. (2015) proposed two new indexes from the Poincare Plot that should help for a better understanding of the autonomic balance through HRV analysis: (i) Stress Score (SS), calculated as 1,000 × 1/SD2, as a direct assessment of sympathetic activity, and (ii) Sympathetic/Parasympathetic Ratio (S/PS), calculated as SS/SD1, as an indicator of autonomic balance. Naranjo-Orellana et al. (2015) studied the validity of SS and S/PS as HRV measurements and also set normal values for elite soccer players. However, to our knowledge, there are not studies investigating the validity of SS and S/PS as HRV indicators in sedentary healthy adults.

Therefore, we aimed (i) to evaluate the association of SS and S/PS with time domain and frequency domain analysis and (ii) to set the reference values of SS and S/PS in sedentary healthy adults. We hypothesized that high levels of SS and S/PS would be related to lower values of parasympathetic activity outcomes.

## Materials and Methods

### Participants and study design

A total of 156 participants (81 women) aged between 18 and 65 years old participated in the current cross-sectional study. Participants were sedentary (<20 min of physical activity on <3 days/week) and healthy individuals from the province of Granada (Spain). The inclusion criteria were to show a stable body weight over the previous 3 months (changes <5 kg) and to have no chronic metabolic disease, cancer, or any problem that could be aggravated by physical activity. Participants were enrolled in two different randomized control trials, respectively: the FIT-AGEING study (ClinicalTrials.gov ID: NCT03334357) (Amaro-Gahete et al., 2018) and the BEER-HIIT study (ClinicalTrials.gov ID: NCT03660579) which mainly aimed to investigate the effects of different exercise training modalities on health-related parameters. Both projects were approved by the Ethics Committees on Human Research of both the government authority and the University of Granada (0838-N-2017 and 321/CEIH/2017) and followed the principles of the last revised Declaration of Helsinki (7th revision of October 2013) (World Medical Association, 2013). All participants signed a written informed consent and agreed to transfer their scientific data for other scientific purposes or research studies.

Evaluation tests were performed at the Sports and Health Research Centre (CIDS), University of Granada (Spain). Participants came to our laboratory between 7.00 and 11.00 a.m. meeting the following pre-conditions: (i) normal sleep the night before, (ii) abstaining from alcohol intake and drugs or stimulant consumption, including coffee and others stimulant 24 h before, and (iii) avoiding strenuous physical exercise during the two days before the test. The environmental conditions were standardized (22–23 °C).

### HRV analysis

(i) HR assessment: The assessment of HRV was carried out in a supine position. Polar RS800CX (Polar Electro, Kempele, Finland) was used to evaluate HRV (R-R series activated). Heart activity was recorded for 15 min, and participants were instructed not to talk or move and to relax as much as possible. The participants were in a supine position for 5 min prior to the start of the HRV test.

(ii) HRV analysis: Data were downloaded by the Polar Pro Trainer 5^®^ software. HRV files were analysed with the Kubios HRV Standard^®^ (University of Eastern Finland, Kuopio, Finland) software (Tarvainen et al., 2014). Artefacts of the recordings were excluded eliminating RR intervals which differed more than 25% from the previous and the subsequent RR intervals and were replaced with conventional spline interpolation following the methodology described by previous studies and applying the medium filter provided by the Kubios HRV Standard^®^ (Shaffer & Ginsberg, 2017). The smoothness prior approach with a Lambda value of 500 was used to remove not valid low-frequency baseline trend components (Tarvainen et al., 2014).

(iii) Time-domain frequency-domain and non-linear domain analysis: Results from the Time-Domain methods (SDNN, RMSSD and PNN50), the Frequency-Domain methods (HF, LF and LF/HF ratio) and the non-linear analyses (SD1, SD2 and SD2/SD1 ratio) were obtained by the HRV Kubios software following standard procedures (Electrophysiology TF of the ES, 1996). SS and S/SP were also calculated.

### Statistical analyses

Data are expressed as mean ± standard deviation. Mann-Whitney tests were carried out in order to assess the homogeneity of genders. Both linear and non-linear regression models were built to study the relation of SD1, SD2, SS and S/PS with variables traditionally used for HRV analysis. The relationship of SS and S/PS with age was also assessed. Normality of SS and S/PS was checked (i.e., Shapiro–Wilk test, visual check of histograms and Q–Q plots) and Student Unpaired Test was used to look for differences between genders in SS and S/PS. Effect Size was calculated with Hedges’ G correction formula, categorizing the results as trivial (<0.2), small (0.2–0.5), medium (0.5–0.8) and large (>0.8) based on a modification by Sullivan & Feinn (2012). Acceptance intervals categorized by age group (18–25, 35–45, 45–55 and 55–65 years old) were calculated setting confidence (1−α) at 0.95 and proportion of observations within the interval (π) at 0.90.

Statistical analyses were performed using the Statistical Package IBM Statistical Package for Social Sciences (IBM Corporation, Endicott, NY, USA, Released 2017. IBM SPSS Statistics for Windows, Version 25.0. Armonk, NY, USA: IBM Corporation) and the Software Graph Pad Prism version 7.0 (Graph Pad Software Inc., San Diego, CA, USA). Type I error (α) was set at 0.05.

## Results

Descriptive parameters of the study sample can be found in Table 1. Women showed lower Low/High Frequency Ratio and SD2/SD1 ratio than men (all *P* < 0.05).

**Table 1:** Descriptive values for all variables of the study.

	All (*n* = 156)	Men (*n* = 75)	Women (*n* = 81)
Age (years)	38.4 ± 15.57	39.1 ± 15.60	37.8 ± 15.6
Weight (kg)	72.3 ± 16.7	85.2 ± 17.6	64.2 ± 10.9
Height (m)	1.68 ± 0.09	1.76 ± 0.06	1.62 ± 0.05
Heart rate (bpm)	66.2 ± 9.97	65.1 ± 10.15	67.4 ± 9.72
SDNN (ms)	41.3 ± 21.49	42.9 ± 22.96	39.87 ± 20.06
RMSSD (ms)	43.2 ± 27.57	43.4 ± 29.42	43.13 ± 25.9
PNN50 (%)	21.3 ± 21.15	21.4 ± 21.24	21.23 ± 21.21
High frequency (ms^2^)	941 ± 1225	945 ± 1454	937 ± 971
Low frequency (ms^2^)	941 ± 1099	1076 ± 1259	817 ± 919
Low/high frequency ratio	1.6 ± 1.96	1.8 ± 1.63*	1.5 ± 2.24*
SD1 (ms)	30.6 ± 19.53	30.7 ± 20.84	30.6 ± 18.4
SD2 (ms)	49.0 ± 24.56	51.3 ± 26.4	46.9 ± 22.7
SD2/SD1 ratio	1.7 ± 0.59	1.91 ± 0.67*	1.7 ± 0.47*
Stress score (ms)	26.3 ± 14.05	25.9 ± 15.24	26.7 ± 12.91
Sympathetic/parasympathetic ratio	1.6 ± 2.11	1.8 ± 2.65	1.5 ± 1.43

**Notes:**

* *P* < 0.05 between gender on Mann–Whitney Test.

Data are presented as Mean ± Standard Deviation. Abbreviations: SDNN, standard deviation of RR intervals; RMSSD, square root of the mean squared differences between successive RR intervals; PNN50, percentage of successive RR interval pairs that differ more than 50 ms; SD1, standard deviation of Poincare plot orthogonal to the line-of-identity; SD2, standard deviation of Poincare plot along the line-of-identity; bpm, beats per minute; ms, milliseconds; ms^2^, milliseconds square; %, percentage.

**Figure 1:**
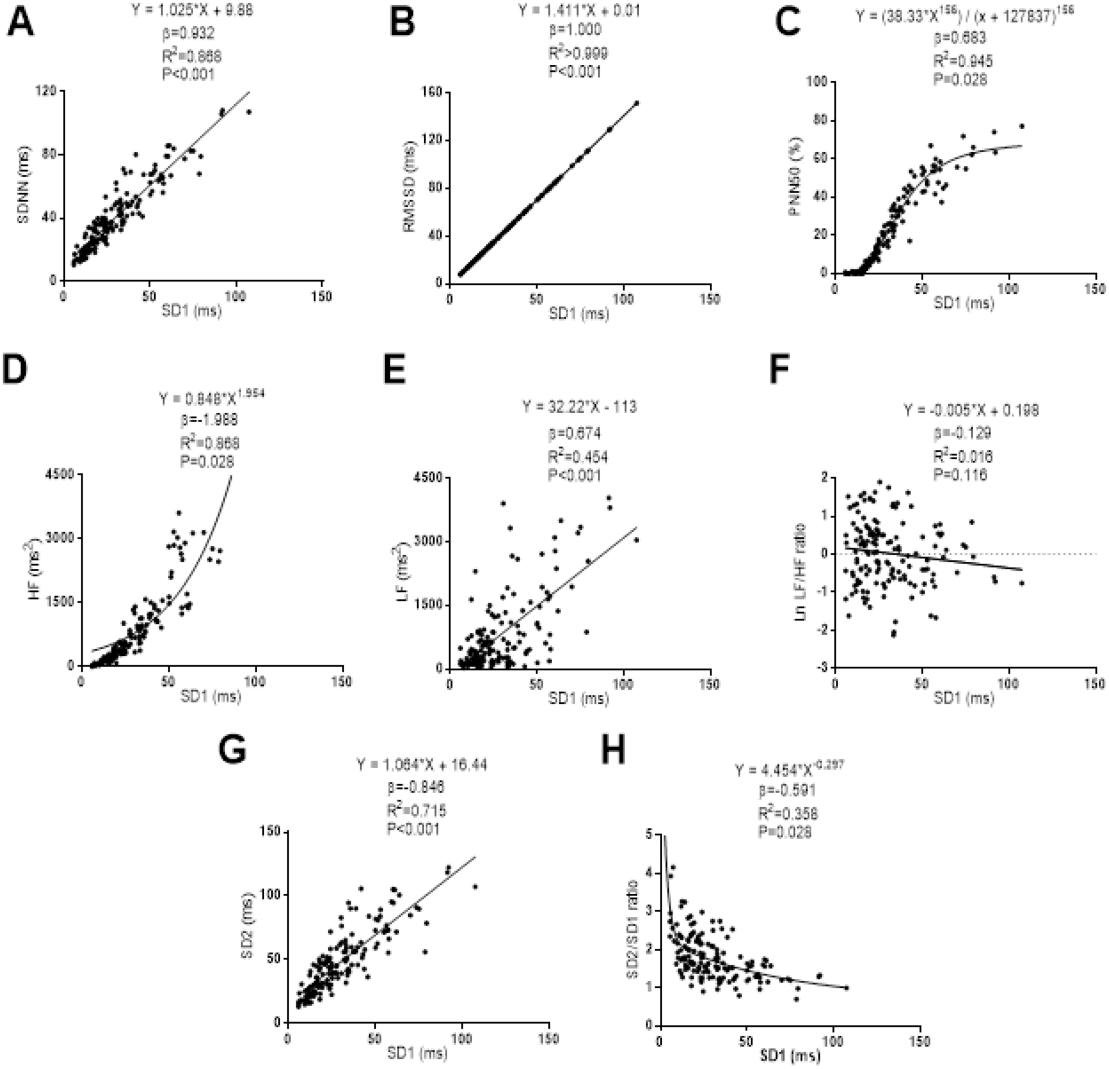
Association between SD1 and SDNN (A), RMSSD (B), PNN50 (C), high frequency (D), low frequency (E), ratio low-high frequency (F), SD2 (G) and ratio SD2/SD1 (H). β (standardized regression coefficient), R2 (determination coefficient) and P (level of significance) from simple linear regression and non-linear regression analysis respectively. Abbreviations: SDNN, standard deviation of RR intervals; RMSSD, root mean square of successive differences; PNN50, percentage of successive intervals that differs more than 50 ms; SD1, standard deviation of Poincare plot orthogonal to the line-of-identity; SD2, standard deviation of Poincare plot along the line-of-identity; ms, milliseconds; %, percentage and ms^2^, milliseconds square.

The associations of SD1 with the Time-Domain, Frequency-Domain and non-linear analyses of HRV are presented in Fig. 1. Linear regression models were established between SD1 with SDNN (β = 0.932; *R*^2^ = 0.868; *P* < 0.001), RMSSD (β = 1.000; *R*^2^ > 0.999; *P* < 0.001), Low Frequency (β = 0.674; *R*^2^ = 0.454; *P* < 0.001) and SD2 (β = 0.846; *R*^2^ = 0.715; *P* < 0.001). Non-linear regression models were built between SD1 and PNN50, High Frequency and Ratio SD2/SD1. SD1 showed a sigmoid relationship with PNN50 (β = 0.683; *R*^2^ = 0.945; *P* = 0.028), while a positive power relationship was observed between SD1 with High Frequency (β = 1.988; *R*^2^ = 0.858; *P* = 0.028). SD1 showed a negative power relationship with Ratio SD2/SD1 (β = −0.591; *R*^2^ = 0.358; *P* = 0.028). We did not observe a significant relationship between SD1 and Low/High Frequency Ratio (β = −0.129; *R*^2^ = 0.016; *P* = 0.116).

**Figure 2:**
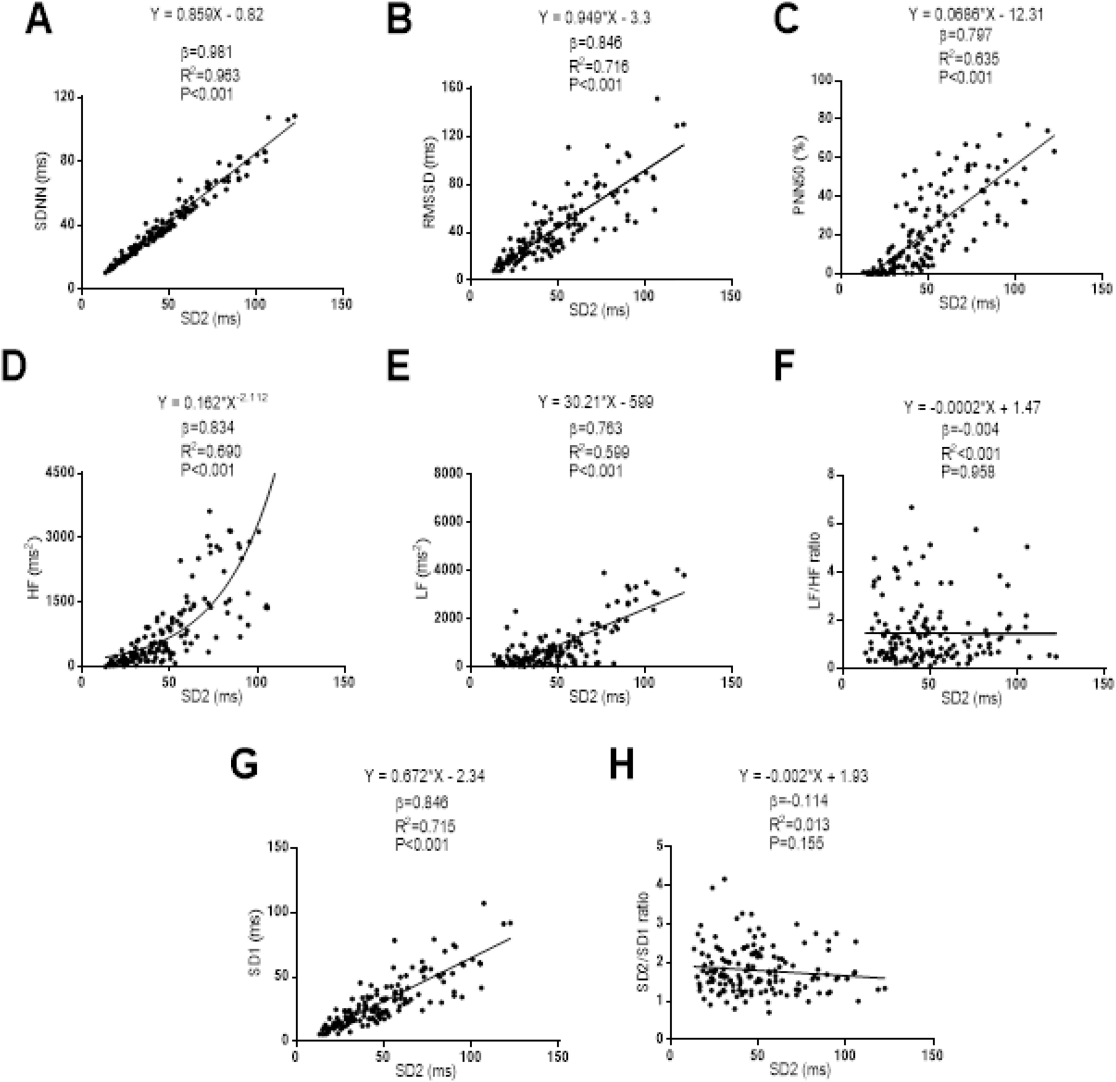
Association between SD2 and SDNN (A), RMSSD (B), PNN50 (C), high frequency (D), low frequency (E), ratio low–high frequency (F), SD1 (G) and ratio SD2/SD1 (H). β (standardized regression coefficient), R2 (determination coefficient) and *P* (level of significance) from simple linear regression and non-linear regression analysis respectively. Abbreviations: SDNN, standard deviation of RR intervals; RMSSD, root mean square of successive differences; PNN50, percentage of successive intervals that differs more than 50 ms; SD1, standard deviation of Poincare plot orthogonal to the line-of-identity; SD2, standard deviation of Poincare plot along the line-of-identity; ms, milliseconds; %, percentage and ms^2^, milliseconds square.

The relationships between SD2 with the Time-Domain, Frequency-Domain and non-linear analysis outcomes can be seen in Fig. 2. A linear regression model was established between SD2 with SDNN (β = 0.968; *R*^2^ = 0.963; *P* < 0.001), RMSSD (β = 0.846; *R*^2^ = 0.716; *P* < 0.001), PNN50 (β = 0.797; *R*^2^ = 0.635; *P* < 0.001), Low Frequency (β = 0.763; *R*^2^ = 0.599; *P* < 0.001), and SD1 (β = 0.846; *R*^2^ = 0.715; *P* < 0.001). A non-linear regression model was established between SD2 and High Frequency showing a positive power relationship between them (β = 0.834; *R*^2^ = 0.690; *P* < 0.001). There was no relationship between SD2 and Low/High Frequency ratio (β = −0.004; *R*^2^ < 0.001; *P* = 0.958), as well as between SD2 with SD2/SD1 ratio (β = −0.114; *R*^2^ = 0.014; *P* = 0.156).

**Figure 3:**
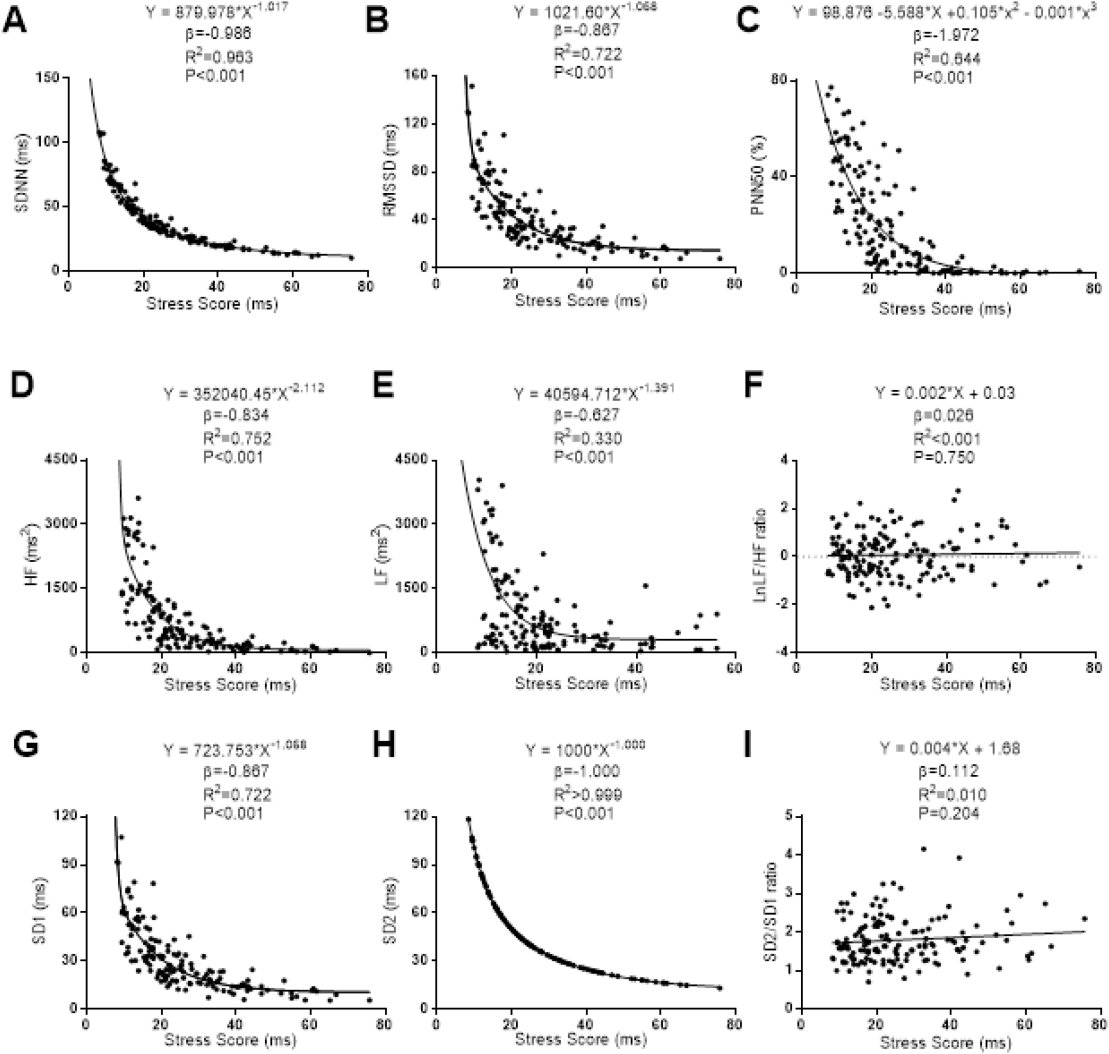
Association between stress score and SDNN (A), RMSSD (B), PNN50 (C), high frequency (D), low frequency (E), ratio low–high frequency (F), SD1 (G), SD2 (H) and ratio SD2/SD1 (I). β (standardized regression coefficient), R2 (determination coefficient) and *P* (level of significance) from simple linear regression and non-linear regression analysis respectively. Abbreviations: SDNN, standard deviation of RR intervals; RMSSD, root mean square of successive differences; PNN50, percentage of successive intervals that differs more than 50 ms; SD1, standard deviation of Poincare plot orthogonal to the line-of-identity; SD2, standard deviation of Poincare plot along the line-of-identity; Ln, Napierian logarithm; ms, milliseconds; %, percentage and ms^2^, milliseconds square.

The associations of SS with the Time-Domain, Frequency-Domain and non-linear analyses of HRV are presented in Fig. 3. Non-linear regression models were stablished between SS with SDNN, RMSSD, high frequency, low frequency, SD1 and SD2. SS showed a non-linear negative power-law relationship with SDNN (β = −0.969; *R*^2^ = 0.963; *P* < 0.001), RMSSD (β = −0.867; *R*^2^ = 0.722; *P* < 0.001), high frequency (β = −0.834; *R*^2^ = 0.752; *P* =< 0.001), Low Frequency (β = −0.627; *R*^2^ = 0.330; *P* < 0.001), SD1 (β = −0.867; *R*^2^ = 0.722; *P* < 0.001) and SD2 (β = −1.000; *R*^2^ > 0.999; *P* < 0.001). There was observed a negative cubic relationship between SS with PNN50 (β = −1.972; *R*^2^ = 0.644; *P* < 0.001). A linear regression model was conducted between SS with Ratio Low/High Frequency (β = 0.026; *R*^2^ < 0.001; *P* = 0.750). No linear association was observed between SS and Ratio SD2/SD1 (β = 0.112; *R*^2^ = 0.010; *P* = 0.204).

**Figure 4:**
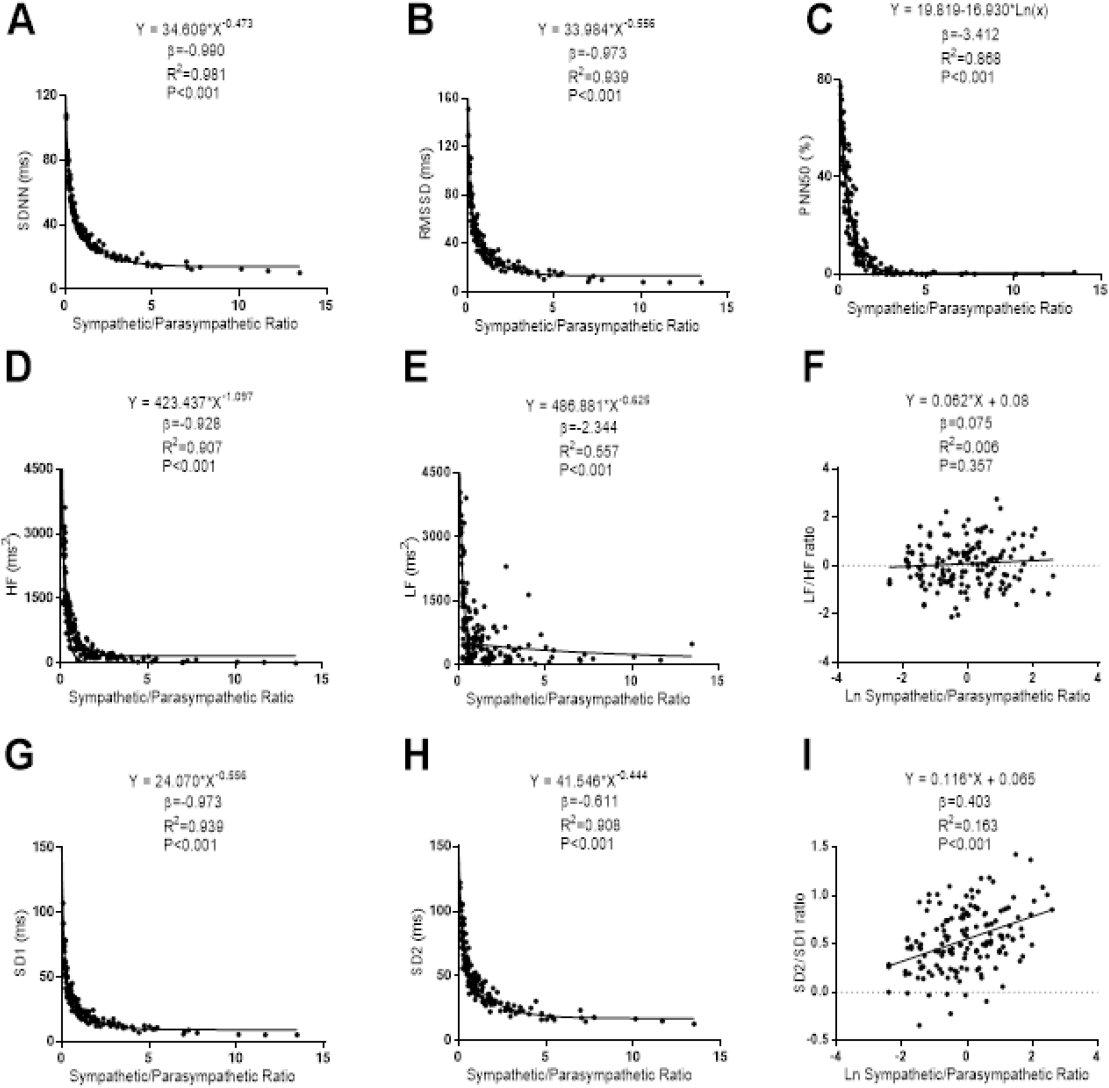
Association between stress score and SDNN (A), RMSSD (B), PNN50 (C), high frequency (D), low frequency (E), ratio low–high frequency (F), SD1 (G), SD2 (H) and ratio SD2/SD1 (I). β (standardized regression coefficient), R2 (determination coefficient) and *P* (level of significance) from simple linear regression and non-linear regression analysis respectively. Abbreviations: SDNN, standard deviation of RR intervals; RMSSD, root mean square of successive differences; PNN50, percentage of successive intervals that differs more than 50 ms; SD1, standard deviation of Poincare plot orthogonal to the line-of-identity; SD2, standard deviation of Poincare plot along the line-of-identity; ms, milliseconds; %, percentage and ms^2^, milliseconds square; Ln, Napierian logarithm.

The associations of S/PS with the Time-Domain, Frequency-Domain and non-linear analyses of HRV can be seen in Fig. 4. Non-linear power-law regression models were built between S/PS and SDNN (β = −0.990; *R*^2^ = 0.981; *P* < 0.001), RMSSD (β = −0.973; *R*^2^ = 0.939; *P* < 0.001), high frequency (β = −0.928; *R*^2^ = 0.970; *P* < 0.001), low frequency (β = −2.344; *R*^2^ = 0.557; *P* < 0.001), SD1 (β = −0.973; *R*^2^ = 0.939; *P* < 0.001) and SD2 (β = −0.611; *R*^2^ = 0.908; *P* < 0.001). A non-linear negative regression model was built between S/PS and PNN50 (β = −3.412; *R*^2^ = 0.868; *P* < 0.001). A linear regression model was conducted between S/PS and SD2/SD1 (β = 0.075; *R*^2^ = 0.006; *P* < 0.001). We did not find any association between S/PS with Ratio Low/High Frequency (β = 0.075; *R*^2^ = 0.006; *P* < 0.357).

**Figure 5:**
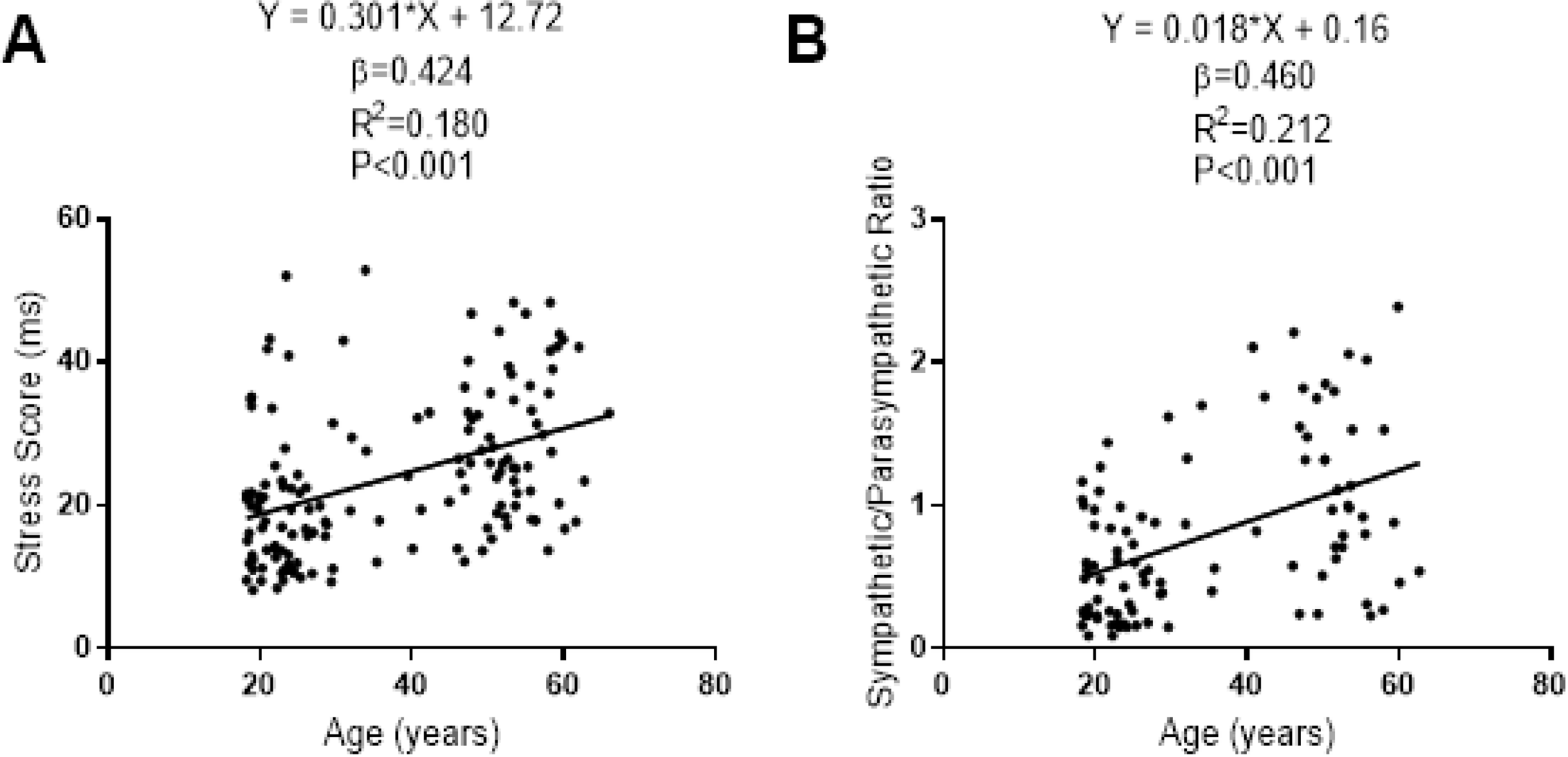
Association between age and stress score (A) and sympathetic/parasympathetic ratio (B) for central 90% of distribution of each dependent variable. β (standardized regression coefficient), R2 (determination coefficient) and *P* (level of significance) from a simple linear regression analysis. Abbreviations: ms, milliseconds.

The associations of SS and S/PS with the participants’ age are presented in Fig. 5. There was a linear positive association of SS and S/PS with the participants’ age (β = 0.424; *R*^2^ = 0.180; *P* < 0.001; β = 0.460; *R*^2^ = 0.212; *P* < 0.001, respectively).

**Figure 6:**
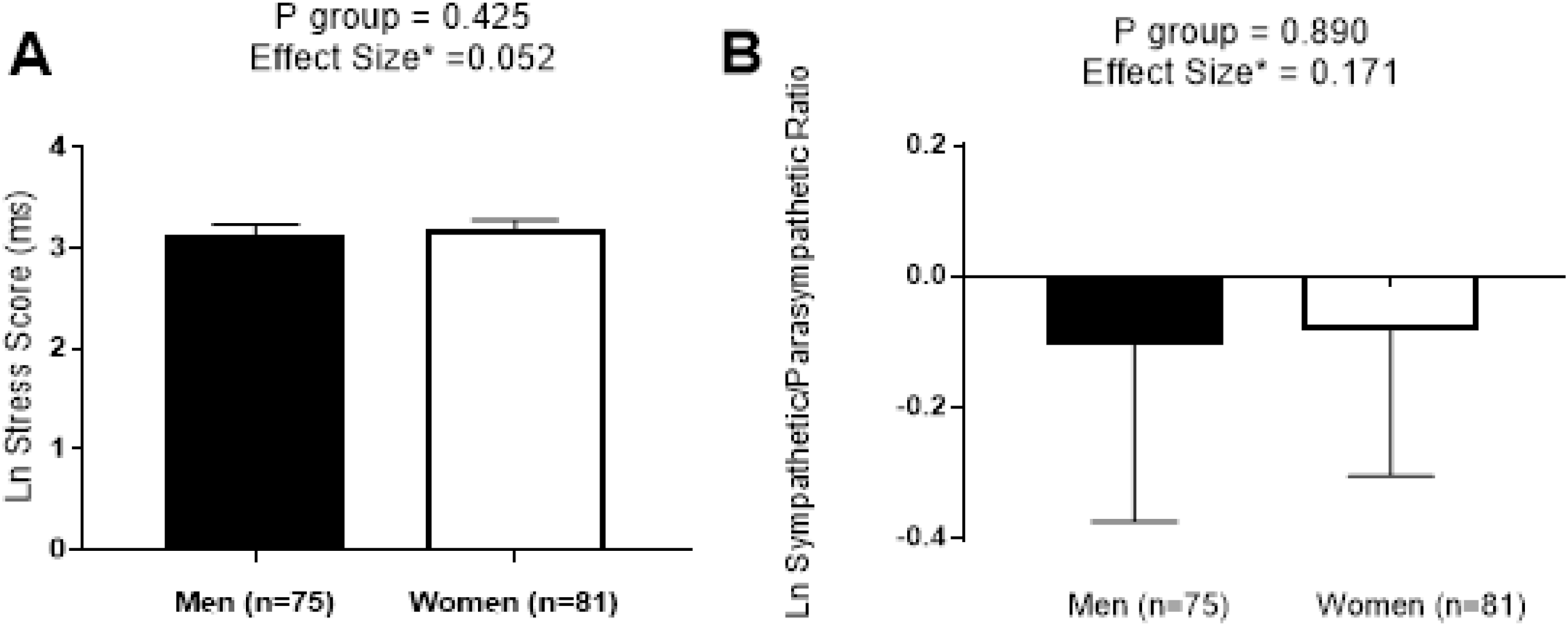
Differences between sexes in stress score (A) and sympathetic/parasympathetic ratio (B). Data are represented as Mean ± 95% CI. Abbreviations: *P*, level of significance calculated by Student *T*-Test; Ln, Napierian logarithm; ms, milliseconds; *, correction of Hedges’ G effect size formula.

We performed a comparative analysis of SS and S/PS normalized by logarithmic transformation between sexes in Fig. 6. No significant differences were found between sexes (all *P* < 0.425), and a trivial effect size was detected in both cases (0.052 and 0.171, respectively).

SS and S/PS acceptance intervals calculated by age groups are presented in Table 2. The acceptance intervals were generally wide in older than younger participants.

**Table 2:** Acceptance intervals for stress score and sympathetic/parasympathetic ratio dividing by age group.

Age groups	Stress score (ms)	Sympathetic/parasympathetic ratio
18–25 years old (*n* = 55)	(6.89; 46.42)	(0.06; 4.12)
25–35 years old (*n* = 20)	(6.62; 55.77)	(0.06; 5.79)
35–45 years old (*n* = 8)	(6.60; 63.35)	(0.07; 8.65)
45–55 years old (*n* = 44)	(12.10; 66.18)	(0.27; 8.28)
55–65 years old (*n* = 28)	(12.16; 89.73)	(0.14; 21.14)

**Note:**

Acceptance intervals were calculated with α = 0.05 and π = 0.90. Abbreviations: *n*, number of participants; ms, milliseconds.

## Discussion

The main results of this study suggested that SS could be considered an excellent indicator of the sympathetic activity, and that S/PS can accurately determine the relationships between the sympathetic and parasympathetic activities of the ANS.

It has been previously reported that SD1 showed a positive relationship with RMSSD, PNN50 and High Frequency, which are related to the parasympathetic activity (Thayer, Yamamoto & Brosschot, 2010), and also with SDNN, which is considered a global HRV marker (Michael, Graham & Davis, 2017). Our findings concurred with those observed in previous investigations suggesting SD1 as a good parasympathetic activity marker (Tulppo et al., 1996; Hoshi et al., 2013).

There is controversial evidence about the physiological implications of SD2 as an HRV outcome. Previous studies have considered SD2 as an inverse indicator of the sympathetic activity (Naranjo-Orellana et al., 2015). Considering the almost perfect negative correlation between SD2 and SDNN in addition to a strong negative correlation of SD2 with RMSSD, PNN50 and High Frequency observed in our study, our findings support the notion that SD2 can be considered an excellent index of the inverse sympathetic activity (Naranjo-Orellana et al., 2015).

SS showed a strong negative relationship with well-known parasympathetic activity markers (i.e., SDNN, RMSSD, PNN50, high frequency and SD1) (Thayer, Yamamoto & Brosschot, 2010). Assuming the relationships between the sympathetic and parasympathetic activity of the ANS (Almeida-Santos et al., 2016; Sztajzel, 2004), SS could be considered a good indicator of sympathetic activity. Our results concurred with those reported by a previous study developed in professional soccer players (Naranjo-Orellana et al., 2015) observing slightly higher values of SS in our study. These differences could be explained because our participants were sedentary healthy adults, and a predominance of sympathetic activity related to age and physical fitness level has been previously described (Valentini & Parati, 2009; Lanfranchi & Somers, 2002) compared with professional soccer players (Naranjo-Orellana et al., 2015).

S/PS has been proposed as an indicator of autonomic balance (Naranjo-Orellana et al., 2015). The HRV outcomes proposed to assess the relationship between sympathetic and parasympathetic activity of the ANS are (i) Low/High Frequency Ratio (Montano et al., 1994) and (ii) SD2/SD1 ratio (Guzik et al., 2007). On the one hand, we did not find any significant relationship between S/PS and Low/High Frequency ratio, thus, taking together our finding and those obtained by a previous study (Michael, Graham & Davis, 2017), Low Frequency should not be considered a good sympathetic activity indicator. Consequently, the Low/High Frequency ratio should not be considered a valid index to analyse the relationship between the sympathetic and parasympathetic activities (Billman, 2013; Medeiros, Michael & Boullosa, 2018). On the other hand, Naranjo-Orellana et al. (2015) suggested that SD2/SD1 is not a good measurement of sympathetic and parasympathetic function, since it would rather provide information about the inverse of the sympathetic function and the parasympathetic function (Naranjo-Orellana et al., 2015). In this sense, the relationship of S/PS behaviour with the time-domain HRV outcomes observed in our study concurred with the findings reported by Naranjo-Orellana et al. (2015).

We found a trivial effect size in SS and S/PS between sexes that concurred with the results reported in a recent meta-analysis in which a small effect size between sexes in SD1 and SD2 was observed (Koenig & Thayer, 2016). The aging process is accompanied by a loss of global autonomic regulation and a prevalence of the sympathetic activity of the ANS according to Almeida-Santos et al. (2016). Therefore, the positive and strong linear relationship between SS and S/PS with age supports the use of both parameters as an excellent sympathetic activity and ANS balance markers.

We divided our participants cohort into five age groups and we calculated acceptance intervals for each one. The normal values of SS and S/PS were higher in older participants, which is consistent with the age-related effects on ANS balance previously described. Naranjo-Orellana et al. (2015) set reference values for SS and S/PS of professional soccer players through percentiles. However, we calculated acceptance intervals for our study cohorts. Acceptance intervals are an inference statistical method that shows up tolerance limits of each variable (calculated in function of the average, standard deviation and K factor) determined on the base of the sample size, confidence (α), and the proportion of observations within the interval (π) (Martín-Andrés & Luna del Castillo, 2004). Therefore, the use of acceptance intervals allows us to give reference values more accurately than using percentiles.

The results of the current study should be considered cautiously, since some limitations are present. We conducted the FIT-AGEING and the HIIT-BEER projects in two different seasons (autumn vs. winter). Kristal-Boneh et al. (2000) reported a variation coefficient lower to 3% over SDNN, RMSSD and PNN50 between different seasons of the year (winter vs. summer), but to our knowledge, there are no studies that have investigated the effects of seasonal variation on non-linear HRV outcomes in sedentary healthy adults. Moreover, these findings cannot be extended to older and/or younger individuals.

## Conclusions

In summary, the results of our study support the use of SS as a sympathetic activity marker and S/PS as an indicator of the sympathetic and parasympathetic activities of the ANS in sedentary healthy adults. These findings have important clinical implications since the determination of SS and S/PS by HRV assessment seems to be a simply, cheap and valuable method to study both the sympathetic and parasympathetic activities of the ANS in sedentary but healthy adults.

## Supplemental Information

10.7717/peerj.10178/supp-1 Supplemental Information 1 Raw data.

## Additional Information and Declarations

### Data Availability

The following information was supplied regarding data availability: The raw measurements are available as a Supplemental File.
